# Adult mortality or morbidity is not increased in childhood-onset growth hormone deficient patients who received pediatric GH treatment: an analysis of the Hypopituitary Control and Complications Study (HypoCCS)

**DOI:** 10.1007/s11102-013-0529-6

**Published:** 2013-10-12

**Authors:** Daojun Mo, Dana Sue Hardin, Eva Marie Erfurth, Shlomo Melmed

**Affiliations:** 1 Lilly Research Laboratories, Eli Lilly and Company, Lilly Corporate Center, Indianapolis, IN 46285 USA; 2 Department of Endocrinology, Skånes University Hospital, Lund, Sweden; 3 Pituitary Center, Department of Medicine, Cedars-Sinai Medical Center, Los Angeles, CA USA

**Keywords:** Growth hormone deficiency, Mortality, Morbidity, GH treatment, HypoCCS, SAGhE

## Abstract

**Background:**

The French Safety and Appropriateness of Growth Hormone treatments in Europe (SAGhE) cohort has raised concern of increased mortality risk during follow-up into adulthood in certain patients who had received growth hormone (GH) treatment during childhood. The Hypopituitary Control and Complications Study monitored mortality and morbidity of adult GH-deficient patients including those with childhood-onset GH deficiency (COGHD) who received GH treatment as children.

**Purpose:**

Evaluate risk of mortality, cancer, myocardial infarction (MI) and stroke in a prospective observational study.

**Methods:**

COGHD patients [n = 1,204, including 389 diagnosed with idiopathic COGHD (ICOGHD)] had received pediatric GH treatment. Standardized mortality ratios (SMRs), and cancer standardized incidence ratios (SIRs) in patients without a prior cancer were estimated relative to reference populations. Crude incidence rates were estimated for MI and stroke.

**Results:**

No increased mortality or cancer incidence was observed, as compared with reference populations, during a follow-up of 3.7 ± 3.3 years (mean ± SD). The overall SMR for COGHD was 1.14 [95 % confidence interval (CI) 0.55–2.10], and for ICOGHD, 0.33 (0.01–1.84). The overall cancer SIR for COGHD was 0.27 (0.01–1.50), and for ICOGHD, 0.00 (0.00–2.45). No incident case of MI was reported. The crude stroke incidence rate [181.3 per 100,000 person-years] in COGHD patients was consistent with the rates reported in reference populations. No incident case of stroke was identified in ICOGHD patients who are presumed to have no increased stroke risk factors.

**Conclusions:**

The results indicate no increased risk of mortality or incidence of cancer, stroke, or MI in adult GH-deficient patients who had previously received pediatric GH treatment.

## Introduction

The reported results of the study of Safety and Appropriateness of Growth Hormone treatments in Europe (SAGhE) raised concern regarding the long-term safety of recombinant growth hormone (GH) therapy [1]. The study was a retrospective evaluation of follow up on approximately 7,000 adults who were treated with recombinant GH during childhood for idiopathic isolated GH deficiency, neurosecretory GH dysfunction, idiopathic short stature and born short for gestational age. These patients, presumed to be at no increased risk, were found to have an increased all-cause mortality compared with the French reference population [standardized mortality ratio (SMR) = 1.33]. Furthermore, the study reported increased mortality due to diseases of the circulatory system (SMR = 3.07), subarachnoid intracerebral hemorrhage (SMR = 6.66), and bone tumor (SMR = 5.00). The mean period between start of childhood GH therapy and the time of census or death was approximately 17 years. In contrast, the SAGhE results from Sweden, Belgium, and the Netherlands did not demonstrate any death from cancer or cerebrovascular disease in approximately 2,500 patients studied [2]. Thus, the safety of long-term GH treatment during childhood remains inconclusive [3, 4].

The Hypopituitary Control and Complications Study (HypoCCS), a global postmarketing surveillance study conducted by Eli Lilly and Company, monitored clinical outcomes of adult GH-deficient (GHD) patients [5]. Multiple safety-related HypoCCS analyses have been published [6–10], and the HypoCCS GHD patients included subjects with childhood-onset GH-deficiency (COGHD) who had received GH treatment during childhood, similar to the subjects in SAGhE. When the French SAGhE data were published, the HypoCCS database included 10,190 subjects who had been enrolled from the United States, Canada, and 14 European countries during the period 1995–2010. Of these, 1,024 had a diagnosis of COGHD and a history of prior pediatric GH treatment. These COGHD patients were followed for an average of 3.7 years. Given the uncertainty regarding long-term safety of GH treatment, we employed the HypoCCS database to assess mortality and the incidence rates for cancer, myocardial infarction (MI), and stroke in adult GH-deficient patients who had received pediatric GH treatment.

## Subjects and methods

### Study population

Subjects enrolled in HypoCCS met the adult GH deficiency indication for Humatrope [somatropin (rDNA origin) for injection; Eli Lilly and Co, Indianapolis, IN, USA], according to the prescribing regulation for each country. Subjects had an established diagnosis of adult GH deficiency, either alone or with multiple pituitary hormone deficiencies, as determined by clinical history and biochemical testing [5]. The diagnostic approach used and the decision to administer GH was at the discretion of the investigating site physician. It is unknown whether the patients primed with sex steroids for the diagnostic tests. Subjects were not eligible for enrollment if they had active or unresolved contraindications to GH therapy, including evidence or suspicion of active malignancy, evidence of ongoing pituitary or other intracranial tumor activity, pregnancy, or breastfeeding. HypoCCS was conducted in accordance with the Declaration of Helsinki guidelines and all applicable regulatory requirements in the respective participating countries. Ethical review board approval and written consent for data collection, electronic processing, and publication were obtained in accordance with respective national laws.

For this analysis, we studied the 1,204 childhood-onset GHD patients who were reported as having received pediatric GH treatment and had available information on age, gender, and at least one follow-up visit after study entry (the COGHD group). Of these, 1,086 received GH treatment as adults. Subjects with idiopathic COGHD (the ICOGHD group) (n = 389), a subgroup of the COGHD group presumed to be at no increased risk of mortality, cancer, stroke, or MI, were also analyzed.

### Mortality and incidence of cancer, stroke, and MI

Deaths and adverse events (AEs) were reported from the HypoCCS case report forms (CRFs) for AEs. Lilly pharmacovigilance databases that store spontaneously reported cases were further searched to see if any cases were missed in the HypoCCS CRFs. Any missed cases were added to the report. Causes of death and AEs were coded in *Medical Dictionary for Regulatory Activities* (MedDRA) terms (Version 14.0). Subjects with stroke, MI, and cancer were ascertained in a comprehensive review process. For case ascertainment of stroke, MedDRA preferred terms indicative of stroke were selected from the central nervous system hemorrhages and cerebrovascular conditions Standardized MedDRA Query (SMQ), and the patients with these terms were pre-selected. Of the pre-selected patients, incident cases (new cases that occurred after HypoCCS entry) were then identified by comprehensively reviewing CRFs for patient demographics, pre-existing conditions and comorbidities, historical diagnoses, physical examinations, laboratory measures, somatropin dosing, concomitant medications, previous treatments, CRF comment pages, and AE narratives [6, 7]. Cases of subarachnoid hemorrhage due to trauma were not accounted for as stroke cases according to prior recommendation [11]. Similarly, for case ascertainment of MI and cancer, the MedDRA preferred terms indicative of the diagnoses of MI and cancers were selected from the MI SMQ and the Malignancies SMQ, respectively. After pre-selecting the patients with these terms, incident cases of MI and cancer were ascertained by comprehensive review similar to that conducted for stroke. Only incident AE cases were included for analysis. Mean daily GH dose (micrograms per day) received in HypoCCS was estimated from each follow-up visit, excluding the dosing reported during the first 6 months of treatment.

### Statistical analysis

For baseline demographics and clinical data, number (N) and percentage (%) were summarized for categorical variables (e.g., gender); mean and SD were summarized for continuous variables (e.g., age).

The crude (unadjusted) incidence rate for each AE of interest or the crude (unadjusted) mortality rate was calculated by dividing the number of new cases observed during the study period by the total person-years at risk among patients with no history of the respective AE at HypoCCS entry. For new cases, person-years were defined as intervals between HypoCCS entry and the event dates. For the remainder of the patients, person-years were defined as intervals between the HypoCCS entry and the last visit date recorded in the database.

To compare the observed results in HypoCCS and the general population, standardized mortality ratios (SMRs) and standardized incidence ratios (SIRs) for de novo cancer in patients without a prior reported cancer were estimated by computing a ratio between the number of events observed and that expected. The expected incidence was determined using gender- and age-specific incidence rates for the general population in the United States and other countries participating in HypoCCS. Observed and expected counts for each country were obtained by sum of the gender- and age-specific strata for that respective country. An estimate of the overall SIR or SMR was calculated using pooled results from all countries that participated in HypoCCS, including those with no AEs reported. 95 % confidence intervals (CIs) for the results from the data following a Poisson distribution are presented in all instances [12]. US reference mortality rates were obtained from the Centers for Disease Control and Prevention [13], and non-US reference mortality rates from the World Health Organization [14]. US reference cancer incidence rates were derived from SEER [15], and others from GLOBOCAN [16]. The country-specific SMR or SIR was also reported. Because for every additional country analysis, there is an increased chance of type I error (error of falsely rejecting null hypothesis that there is no increased risk) [17], an observed significant result may be prone to be a false discovery. However, for purpose of safety surveillance, this type I error was not adjusted. Thus, without this adjustment, we used stringently conservative methods in reporting the comparison between the HypoCCS and each of its reference populations.

Because population-based reference data for MI and stroke were not readily available for estimating SIRs, only crude incidence rates were computed for MI and stroke for HypoCCS patients and are presented along with published reports [18, 19].

## Results

### Characteristics of COGHD and ICOGHD groups

One thousand two hundred and four childhood-onset GHD patients were identified in the COGHD group who had been treated with GH during childhood and had available information on age, gender, and follow-up (Table 1). This group had a mean age of 26.8 years, 37 % were female, and mean follow up was 3.7 years. The mean GH dose reported in HypoCCS was 540 μg/day. The reported starting age for GH administration in childhood was 11.3 years. The mean durations of GH therapy before the age of 18 years, the age of 21 years, and HypoCCS entry were 6.6, 7.1, and 7.5 years, respectively. Of this group, 1,086 patients (90.2 % of 1,204) received GH therapy as adults. Causes of GH deficiency included history of craniopharyngioma (20.9 %), history of other intracranial tumors (15.3 %), congenital defects (8.9 %), empty sella (5.9 %), and idiopathic (32.3 %). About 30 % of patients were from the United States, 15 % from Italy, 9 % from Germany, 9 % from Spain and 8 % from Japan and the remaining 29 % from Canada and 11 European countries. In the ICOGHD group, 389 patients were identified who had a longer mean follow-up duration (4.2 years for ICOGHD vs. 3.7 for COGHD) and an earlier starting age of GH therapy (10 years vs. 11.3).

**Table 1:** Characteristics of COGHD and ICOGHD Groups

Characteristic	COGHD (N = 1,204)	ICOGHD (N = 389)
Age (years) at HypoCCS entry, mean (SD)	26.8 (8.6)	27.6 (8.8)
Gender (female), n (%)	449 (37.3)	131 (33.7)
Total follow-up (years) in HypoCCS mean (SD)	3.7 (3.3)	4.2 (3.6)
Mean GH dose (μg/day) for GH treated in HypoCCS, mean (SD)	539.4 (333.2)	538.9 (298.9)
Reported age of starting GH therapy, mean (SD)	11.3 (7.2)	10.0 (6.1)
GH therapy duration before age 18 years, mean (SD)	6.6 (4.2)	7.2 (4.0)
GH therapy duration before age 21 years, mean (SD)	7.1 (4.5)	7.7 (4.4)
GH therapy duration prior to HypoCCS entry, mean (SD)	7.5 (4.3)	8.0 (5.1)
Causes of GHD, n (%)		
Idiopathic GHD	389 (32.3)	389 (100.0)
Craniopharyngioma	252 (20.9)	
Intracranial tumor (other than craniopharyngioma)	184 (15.3)	
Congenital defect, type unknown	107 (8.9)	
Empty sella	71 (5.9)	
Pituitary hemorrhage or infarct	30 (2.5)	
Known genetic defect	23 (1.9)	
Cranial irradiation (causes except hypothalamic/pituitary tumors)	21 (1.7)	
Others	127 (10.6)	

*Intracranial tumor* (*other than craniopharyngioma*) includes germinoma, dysgerminoma, pituitary adenoma, medulloblastoma, astrocytoma glioma and meningioma, Rathke’s cleft cyst, and histiocytosis. *Others* includes pituitary abscess or other infection, head injury, and other causes

*GH* growth hormone, *GHD* growth hormone deficiency, *COGHD group* those patients with childhood-onset GH deficiency who received GH replacement during childhood and had available information on age, gender, and at least one follow-up visit, *HypoCCS* Hypopituitary Control and Complications Study, *ICOGHD group* an idiopathic subgroup of the COGHD patients, *SD* standard deviation

### Mortality

Ten deaths were reported in the COGHD group. The majority of deaths occurred in males (n = 8). All deaths occurred at relatively young ages (n = 5 in the age group 20–29 years, n = 4 in 30–39, and n = 1 in 40–49). Patients died from a variety of causes [3 from infections, 2 from tumors (1 glioblastoma and 1 craniopharyngioma recurrence or meningioma), 2 from suicide, 1 from grand mal seizure, 1 from stroke, and 1 from Creutzfeldt-Jakob disease]. No patient died from MI or cardiac disorders (cardiomyopathy and cardiomegaly). One death was identified in the ICOGHD group and assumed to be at no increased risk. This patient was a male aged 20–29 years who died from Creutzfeldt-Jakob disease. He had previously received human pituitary extracted GH rather than recombinant GH during ages 2–9 years for childhood-onset idiopathic GH deficiency. He had received GH only as a child, never as an adult, but was enrolled in HypoCCS. The investigator reported that the event was related to the childhood exposure to human-derived GH but not to study procedures or adult treatment with GH. Comprehensive review of the other 9 deaths from the COGHD group showed that all had complex underlying conditions, including intracranial tumor and/or surgery, radiotherapy, and hypopituitarism, and thus had increased risk of premature death [6, 19, 20].

Crude mortality rates and SMRs were calculated for each country and for all countries combined to quantitatively evaluate overall mortality rates in the COGHD group (Table 2). A non-statistically significant SMR (1.14, 95 % CI 0.55–2.10) was observed for the relative risk of the mortality after adjusting for age, gender, and country, suggesting no difference in mortality relative to the general population for this group of patients. Country-specific SMRs (or differences from respective general populations) were not statistically significant for any of the countries except for the United Kingdom. The significant UK SMR (10.81, 95 % CI 2.23–31.58) resulted from the reported 3 deaths. All 3 cases had complex underlying medical conditions and were subject to increased risk of premature death. The first patient was a male aged 40–49 years whose death was reported as “possible suicide” from carbon monoxide and alcohol poisoning. The investigator reported the death as unrelated to study drug or protocol procedure. This case was complex; the patient had childhood-onset GH deficiency with a diagnosis of empty pituitary sella and panhypopituitarism, and he had a history of vertebral disk protrusion and prior surgical removal of malignant melanoma. His concomitant medications included thyroxine for hypothyroidism, hydrocortisone for hypoadrenalism, testosterone for hypogonadism, diclofenac for vertebral disc protrusion, alendronate sodium for osteoporosis, and paracetamol/codeine for back pain. The dose of GH administered at HypoCCS enrollment was 600 μg/day. The other two cases were males with history of surgery and/or radiotherapy and of hypopituitarism. One was aged 30–39 years and died from bronchopneumonia and encephalopathy, and the other, aged 20–29 years, died from acute fulminant pancreatitis with known risk factors [obesity (BMI 35 kg/m^2^) and hypertriglyceridemia] for pancreatitis. In both cases, the investigators listed the findings as unrelated to GH treatment or study protocol.

**Table 2:** Crude mortality rate and SMRs by country for COGHD group

Country	N	Follow-up years	Crude mortality (100,000 person-years) (95 % CI)	Observed deaths	Expected deaths	SMR (95 % CI)
Czech Republic	27	141.4	707.2 (17.9–3,940.4)	1	0.299	3.35 (0.08–18.64)
France	125	372.2	806.0 (166.2–2,355.6)	3	0.927	3.24 (0.67–9.46)
Germany	113	505.22	197.9 (5.0–1,102.8)	1	1.12	0.89 (0.02–4.97)
Netherlands	66	474.74	210.6 (5.3–1,173.6)	1	0.779	1.28 (0.03–7.15)
United Kingdom	40	160.81	1,865.5 (384.7–5,451.9)	3	0.278	10.81 (2.23–31.58)
United States	359	1,134.8	88.1 (2.2–491.0)	1	1.931	0.52 (0.01–2.89)
Overall	1,204	4,462.4	224.0 (107.5–412.1)	10	8.751	1.14 (0.55–2.10)

Mortality rate per 100,000 person-years = observed number of deaths × 100,000/total person-years. Expected number of deaths = sum of person-years × incidence rate for each age-gender group. Overall expected number of deaths = sum of expected deaths in each country. Overall observed number of deaths = sum of observed deaths in each country. Data for those countries without a death event are not depicted. However, the total person-years of follow-up and expected number of deaths are included in the overall estimate row. SMR = observed number of deaths/expected number of deaths. 95 % CI is the exact confidence interval based on Poisson distribution. Reference data sources for calculating expected cases: http://wonder.cdc.gov/ for US data and http://apps.who.int/ghodata/ for non-US data

*CI* confidence interval, *COGHD group* those patients with childhood-onset GH deficiency who received GH replacement during childhood and had available information on age, gender, and at least one follow-up visit, *GH* growth hormone, *N* number of patients, *SMR* standardized mortality ratio

A non-statistically significant SMR [0.33 (95 % CI 0.01–1.84)] was observed for the ICOGHD group (Idiopathic COGHD who had received prior pediatric GH treatment), suggesting no increased mortality risk in this group.

### Cancer

In COGHD group, one incident case of cancer was identified in those patients without a prior cancer (n = 1,056) (Table 3), and the case was reported in the mortality analysis. This was a male patient diagnosed with malignant melanoma which was surgically removed on the left upper arm at the age 41. The patient had a history of childhood-onset GH deficiency associated with empty pituitary sella (the concomitant medications of thyroxine, hydrocortisone, testosterone, diclofenac, alendronate sodium, and paracetamol/codeine were listed in the part of mortality results). One year after surgical tumor resection, the patient committed suicide (see previous description in the mortality results). A non-statistically significant SIR (0.27, 95 % CI 0.01–1.50) was observed for the relative risk of the overall incidence of cancer by adjusting for age, gender, and country. No new cancer case occurred in the ICOGHD group.

**Table 3:** Crude incident cancer rate and SIR by country for COGHD group

Country	N	Follow-up years	Crude incidence (100,000 person-years) (95 % CI)	Observed cancer cases	Expected cancer cases	SMR (95 % CI)
United Kingdom	31	127.2	786.3 (19.9–4,381.0)	1	0.119	8.37 (0.21–46.6)
Overall	1,056	3,965.4	25.2 (0.6–140.5)	1	3.718	0.27 (0.01–1.50)

Incidence rate per 100,000 person-years = observed number of incident events × 100,000/total person-years. Expected number of events = sum of person-years × incidence rate for each age-gender group. Overall expected number of events = sum of expected incident events in each country. Overall observed number of events = sum of observed incident events in each country. Data for those countries without an event are depicted. However, the total person-years of follow-up and expected number of deaths are included in the overall estimate row. SIR = observed number of events/expected number of events. 95 % CI is the exact confidence interval based on Poisson distribution. Reference data sources for calculating expected cases: http://seer.cancer.gov/statistics for US data and http://globocan.iarc.fr for non-US data

*CI* confidence interval, *COGHD group* those patients with childhood-onset GH deficiency who received GH replacement during childhood and had available information on age, gender, and at least one follow-up visit, *GH* growth hormone, *N* number of patients, *SIR* standardized incidence ratio

### Myocardial infarction

No incident case of MI was identified in either the COGHD or the ICOGHD group.

### Stroke

In the COGHD group, 8 incident stroke cases (1 hemorrhage, 5 ischemia, and 2 unspecific) were identified. The only incident hemorrhagic stroke (cerebral hemorrhage) occurred in a male patient aged 20–29 with an astrocytoma diagnosed at the age of 2 years and a history of pituitary surgery, radiotherapy, and panhypopituitarism. The 5 incident cases of ischemic stroke (2 cerebral infarctions, 2 lacunar infarctions, and 1 Moyamoya disease) in this group had undergone brain surgery and radiotherapy for brain tumors (4 craniopharyngioma cases and 1 astrocytoma), and also recorded other risks including panhypopituitarism, obesity, and metabolic syndrome. Two incident cases diagnosed as stroke and apoplexy were not specified as hemorrhagic or ischemic. One was reported to be secondary to meningocele surgery, and the other had a history of brain surgery and radiotherapy for craniopharyngioma. None of these strokes was considered related to the GH therapy by the reporting investigators. No stroke was identified in the ICOGHD group.

For the COGHD group, crude incidence rates for hemorrhagic stroke and ischemic stroke were calculated (Table 4) in each participating country and in all countries. The 8 cases resulted in a crude incidence rate of 181.28 (95 % CI 78.26–357.19) per 100,000 person-years.

**Table 4:** Crude incidence of stroke by country for COGHD group

Country	N	Follow-up years	Stroke type	Number of cases	Crude incidence rate (per 100,000 person-years) (95 % CI)
France	121	361.3	Unspecific	1	276.8 (7.0–1,542.0)
Germany	112	502.1	Unspecific	1	199.25 (5.0–1,109.6)
Japan	90	136.6	Ischemia	1	732.2 (18.5–4,079.8)
United Kingdom	40	160.8	Ischemia	1	621.9 (15.7–3,464.7)
United States	356	1,130.9	Hemorrhage	1	88.4 (2.2–492.7)
			Ischemia	3	265.3 (54.7–775.3)
Overall	1,189	4,413.1	Hemorrhage	1	22.7 (0.6–126.3)
			Ischemia	5	113.3 (36.8–264.4)
			Unspecific	2	45.3 (5.5–163.7)
			Overall	8	181.3 (78.3–357.2)

Two cases of subarachnoid hemorrhage due to trauma were excluded from the crude incidence rate estimate according to the stroke study in Minnesota stroke registry methodology (11). Incidence rate per 100,000 person-years = observed number of incident events × 100,000/total person-years. Data for those countries without an event are not depicted. However, the total person-years of follow-up are included in the overall estimate row. 95 % CI is the exact confidence interval based on Poisson distribution

*CI* confidence interval, *COGHD group* those patients with childhood-onset GH deficiency who received GH-replacement during childhood and had available information on age, gender, and at least one follow-up visit, *GH* growth hormone, *N* number of patients

## Discussion

This HypoCCS analysis focused on adult patients who had received prior GH treatment for COGHD during childhood. This allowed for observation of potential increased clinical risk (mortality, cancer, stroke, and MI) during adulthood in COGHD patients who were formerly pediatric patients. No increased mortality was observed in the COGHD group compared with the general population (as measured by an SMR of 1.14, 95 % CI 0.55–2.10) after adjusting for age, gender, and country. Furthermore, increased country-specific mortality was not observed in any of the 17 participating countries except the UK.

It is difficult to attribute this observed increased UK mortality to GH therapy because all three cases had complex underlying conditions and were vulnerable to increased risk of premature death. Additionally, the statistical significance for the higher mortality in the UK could also be a false positive, as a type I error was not adjusted for in the multiple comparisons in 17 countries [17]. For every additional country analyzed, there was an increased chance of type I error due to multiplicity. The further subgroup analysis for the ICOGHD group allowed for observation of a group assumed to be at no increased risk. The only death in the ICOGHD group was due to Creutzfeldt-Jakob disease related to prior use of human cadaveric derived GH product [21]. Furthermore, the observation of no mortality increase for ICOGHD patients in HypoCCS is consistent with the observations in the SAGhE cohorts from Sweden, Belgium, and the Netherlands [2] and that in idiopathic GHD patients from a Danish cohort [22]. Therefore, the result of the mortality analysis does not indicate that recombinant GH during childhood is related to increased mortality in either the COGHD or ICOGHD groups.

There was only one incident cancer case (malignant melanoma) identified in the COGHD group. This case led to a non-significant SIR (0.27, 95 % CI 0.01–1.50). Even with the point estimate close to 0.3, the wide confidence interval made it difficult to draw conclusions of a lower risk of cancer occurrence compared with the general population. It is reassuring that we observed no occurrence of new cancers in the ICOGHD group (the low-risk group). In contrast with the French SAGhE data [1], no incident case of malignant neoplasm of the bone or articular cartilage was observed in the COGHD or ICOGHD subjects in HypoCCS [1]. No case of MI was identified in either the COGHD or ICOGHD groups.

Because appropriate reference data are not available in the literature, it is not possible to calculate stroke SIR in which age, gender, and country factors can be adjusted for when comparing the HypoCCS COGHD group and reference populations. Overall stroke incidences in the general population were reported in the United States, European countries (France, Italy, Lithuania, Spain, Poland, and the United Kingdom), and Japan. They ranged from 101 (male) and 63 (female) per 100,000 person-years in Italy and 189 in the United States, to 239 (male) and 159 (female) in Lithuania [18, 19, 23]. In contrast, the stroke rate in HypoCCS COGHD group was 181.28 (95 % CI 78.26–357.19) per 100,000 person-years. Although the point estimate of stroke incidence in HypoCCS COGHD group appeared to be at the high end of the risk range reported in the literature, it is not possible to draw conclusions favoring increased stroke risk due to the wide confidence intervals resulting from a small sample size, and also due to age and gender which could not be adjusted in this analysis. Importantly, all stroke patients in the COGHD group had at least one of the following risk factors: brain surgery and/or radiotherapy due to brain tumor, panhypopituitarism, obesity and other metabolic risks. Adult GH deficient patients, especially patients harboring organic brain lesions, were at higher risk of cerebrovascular diseases [7, 20, 24] Adult GH deficient patients were reported to exhibit high prevalence of metabolic syndrome, which predisposed them to a high risk of cerebrovascular diseases [9, 10, 25]. In fact, GH deficiency itself is a risk factor: low levels of IGF-I resulting from GH deficiency may also contribute to atherosclerosis and cerebrovascular disease [24]. Nevertheless, even with a relatively small sample size, it is reassuring that we observed no occurrence of stroke among ICOGHD group, since these patients are free from organic brain lesions and with no increased risk of cerebrovascular disease at baseline. Thus, our results do not provide evidence supporting increased risk of stroke in the COGHD patients or ICOGHD group who had previously received pediatric GH treatment.

Although statistical analyses and comprehensive case review do not support evidence for increased risk of mortality, stroke, cancer, and MI in the COGHD patients, the following limitations of this analysis are acknowledged. First, the analyses were impeded by the relatively short observation duration (a mean 3.7 years of adulthood follow-up) and the small sample size (n = 1,204) of the COGHD group (Table 1). The sample size for this HypoCCS analysis is approximately 20 % of the French SAGhE cohort. Second, it is not directly comparable between the French SAGhE cohort and the HypoCCS cohort. The majority of French SAGhE patients (approximate 75 %) were idiopathic isolated GHD (no multiple pituitary hormone deficiencies) [1]. The similar group in HypoCCS was the ICOGHD group which included both isolated GHD and multiple pituitary hormone deficiencies of unknown etiology. Given this difference in etiology of GH deficiency, it is plausible that the HypoCCS ICOGHD group actually reflects a higher risk group. Nevertheless, we found no safety signal. The third limitation is a possible selection bias as the COGHD group comprised those with persistent childhood-onset GH deficient patients [26]. Patients in HypoCCS with COGHD must have been diagnosed by their adult endocrinologist with persistent GH deficiency and chosen to be enrolled into HypoCCS. Childhood-onset GH deficient patients who had known active malignant tumors were excluded from HypoCCS according to the protocol. Accordingly, results of this analysis cannot be extrapolated to all childhood-onset GH deficient patients. However, this analysis enabled unique observation of potential increased risk of mortality/morbidity during adulthood in COGHD patients who were formerly treated with GH as pediatric patients, and who demonstrated persistent GH deficiency during adulthood, and were in need of continued GH replacement therapy during the transition period and adulthood [27]. While the French SAGhE cohort stopped GH therapy at a mean age of 15.1 years [1], 90.2 % of the HypoCCS cohort received GH therapy as adults. Mean duration of GH therapy was 3.9 years in the French SAGhE [1], but longer durations of GH therapy (6.6 years before the age of 18, 7.1 years before the age of 21, and 7.5 years before HypoCCS entry) were reported in the HypoCCS cohort. Because of persistent GH deficiency and entirely different background disease entities (except for ICOGHD) in HypoCCS, the HypoCCS cohort serves as an important complementary cohort for observations that could not be included in the SAGhE study. Importantly, the reported duration of GH therapy prior to HypoCCS had been accumulated mostly before the age of 18 years (i.e., 6.6 years out of 7.5 years), and only approximately 1 further year of GH therapy was reported during a period of 9 years following the age of 18 years to HypoCCS entry (with a mean age of 26.8 years at entry; Table 1). The HypoCCS data indicate that many patients who had received prior GH therapy during childhood did not transition to GH therapy during adulthood beyond the cessation of linear growth. In practice, many patients with childhood onset GH deficiency are not retested for GH deficiency during this transition [27]. These HypoCCS results suggest the need for future education discussion regarding transition therapy. Fourthly, detailed GH dosing information (μg/kg/day) during childhood was not available in HypoCCS for COGHD patients because it was not specified in the study protocol. Approximately 16 years elapsed between the time patients initiated pediatric GH therapy and the time of HypoCCS enrollment (i.e., from starting GH treatment at age 11.3 years to HypoCCS entry at age 26.8 years; Table 1). However, important information on history of AEs (e.g., types of brain tumor) and risk factors (e.g., brain surgery, radiotherapy, and chemotherapy) prior to HypoCCS enrollment was collected, and these factors likely accounted for the occurrence of the observed AEs. Finally, the small number of patients without GH replacement in childhood (n = 65) restricted comparison between GH treated and untreated pediatric patients. The size of the idiopathic childhood onset subgroup of these 65 patients was even smaller (n = 7). Despite the caveats discussed above, this analysis of the HypoCCS database provides an important opportunity to reassess mortality and morbidity of cancer, MI, and stroke in adult GHD patients who had previously received pediatric GH treatment.

In conclusion, the French SAGhE study [1] raised the important question of increased adult mortality after prior GH treatment in childhood. However, results of this surveillance study indicate no increased risk of mortality or incidence of cancer, stroke, or MI in adult GH-deficient patients who had received prior pediatric GH treatment. Importantly, interpretation of the analysis is limited by the selected patient population, a relatively short follow-up duration, and lack of information on pediatric GH doses administered.
